# Quantification of EV-associated miRNA in liquid biopsies for biomarker signature development

**DOI:** 10.20517/evcna.2026.19

**Published:** 2026-06-03

**Authors:** Mia S. C. Yu, Christian Grätz, Dapi Menglin Chiang, Olivier Loudig, Yadong Zheng, Cole Hladik, Bethany Hannafon, Navneet Dogra, Benedikt Kirchner, Michael W. Pfaffl

**Affiliations:** ^1^Division of Animal Physiology and Immunology, School of Life Sciences Weihenstephan, Technical University of Munich, Freising 85354, Germany.; ^2^Data Science in Systems Biology, School of Life Sciences, Technical University of Munich, Freising 85354, Germany.; ^3^Center for Discovery and Innovation, Nutley, NJ 07110, USA.; ^4^Key Laboratory of Applied Technology on Green-Eco-Healthy Animal Husbandry of Zhejiang Province, Zhejiang Provincial Engineering Laboratory for Animal Health Inspection & Internet Technology, Zhejiang International Science and Technology Cooperation Base for Veterinary Medicine and Health Management, China-Australia Joint Laboratory for Animal Health Big Data Analytics, College of Animal Science and Technology & College of Veterinary Medicine of Zhejiang A&F University, Hangzhou 311300, Zhejiang, China.; ^5^Department of Cell Biology, University of Oklahoma Health Campus, Oklahoma City, OK 73104, USA.; ^6^Department of Obstetrics and Gynecology, University of Oklahoma Health Campus, Oklahoma City, OK 73104, USA.; ^7^Stephenson Cancer Center, University of Oklahoma Health Campus, Oklahoma City, OK 73104, USA.; ^8^Department of Genetics and Genomic Sciences, Icahn School of Medicine at Mount Sinai, New York, NY 10029, USA.; ^9^IBM Thomas J Watson Research Center, Yorktown Heights, NY 10598, USA.; ^10^Division of Pediatric Neuro-Oncology, German Cancer Research Center, Heidelberg 69120, Germany.; ^#^Authors contributed equally to this work.

**Keywords:** miRNA, extracellular vesicles, biomarker, liquid biopsy, molecular diagnostics

## Abstract

Extracellular vesicle (EV)-associated microRNAs (miRNAs) are promising minimally invasive biomarkers, as EV encapsulation protects miRNAs from degradation while preserving disease- and cell- type-specific expression patterns across diverse human body fluids. This review summarizes the current understanding of miRNA and EV biogenesis, including the mechanisms governing EV cargo packaging. We further discuss the broad range of EV sources used in liquid biopsy and molecular diagnostics, including blood, urine, saliva, milk, respiratory fluids, and cerebrospinal fluid, highlighting both the diagnostic utility and key pre-analytical challenges associated with EV-miRNA analysis. State-of-the-art EV isolation approaches, including differential ultracentrifugation, size-exclusion chromatography, affinity-based methods, microfluidic platforms, flow cytometry and nanoparticle-based analyses are not isolation methods and are therefore excluded from this comparison. In addition, workflows for EV-associated RNA extraction and quantification, including RNA sequencing and reverse transcription-quantitative polymerase chain reaction-based methodologies, are critically evaluated. Emphasis is placed on challenges related to data quality assessment, normalization strategies, low-input sample analysis, and high sample heterogeneity. This review also summarizes current standardization initiatives, including MISEV, MIQE, EV-TRACK, and EV Task Force biofluid guidelines, emphasizing the importance of rigorous reporting standards, harmonized pre-analytical workflows, and multiparametric normalization strategies for reproducible detection and accurate quantification. Finally, we discuss emerging best practices and unresolved challenges in multivariate modeling, miRNA isoform-based analyses, and *in silico* validation of the target recognition elements and regulatory pathways. An integrated, guideline-based workflow from liquid biopsy collection to clinically actionable EV-miRNA signatures is proposed to facilitate translation of this approach into routine molecular diagnostics.

## INTRODUCTION

### The discovery of microRNAs

MicroRNAs (miRNAs) are small endogenous RNAs with regulatory activity. In humans, a detailed analysis of all annotated mature miRNAs identified lengths ranging from 16 to 27 nucleotides (nt), with an average length of 22 nt. Loss-of-function screening of genes involved in the developmental timing of *Caenorhabditis elegans* (*C. elegans*) led to the discovery of the first miRNA, lin-4, by the Ambros and Ruvkun laboratories^[1,2]^. This miRNA was partially complementary to several sites in the 3’ untranslated region (UTR) of the lin-14 gene. Ruvkun and colleagues further demonstrated that this sequence complementarity was critical for lin-4-mediated regulation of lin-14, which suppressed LIN-14 protein production. Thereafter, a second miRNA, let-7, and its homologs were identified in *C. elegans* and other animals, ranging from arthropods to vertebrates^[3,4]^. It soon became clear that lin-4 and let-7 represented a foundational RNA family, which was named microRNA in 2001^[5]^. To date, miRNAs have been widely reported in plants, viruses, and animals, with very few exceptions, such as *Theileria* spp. and *Saccharomyces cerevisiae*^[6,7]^. In 2024, Victor Ambros and Gary Ruvkun were awarded Nobel Prize in Physiology or Medicine “for the discovery of miRNA and its role in post-transcriptional gene regulation”^[8]^.

miRNAs can be generated through several pathways. In the canonical pathway, RNA polymerase, typically RNA Polymerase II, first transcribes miRNA genes into long primary transcripts, known as pri-miRNA. These transcripts are then cleaved by the RNase III endonuclease Drosha and its cofactors into approximately 70-nt precursor miRNAs, or pre-miRNAs. Drosha cleavage is highly precise and depends on recognition of the characteristic hairpin structure, as well as specific sequence elements and motifs within the pri-miRNA^[9,10]^. Following transport to the cytoplasm by Exportin-5 and Ran-GTP, pre-miRNAs are further processed by Dicer, another RNase III endonuclease, which releases an imperfect miRNA duplex. With the assistance of chaperones and co-chaperones, one strand of the duplex, the mature miRNA of approximately 21 nt, is incorporated into the RNA-induced silencing complex (RISC) along with Argonaute (AGO) and additional proteins. In contrast, the opposite strand is usually degraded immediately. The strand that is incorporated into RISC (the guide strand) is typically the one with less stable base pairing at its 5’ end; thus, for some miRNA loci the 5p strand serves as the guide, whereas for others the 3p strand is retained^[10]^. However, non-canonical miRNAs have alternative biogenesis pathways that bypass Drosha or Dicer, including mirtrons, which are short intron-derived hairpin RNAs, as well as small nucleolar RNA (snoRNA)-derived and transfer RNA (tRNA)-derived miRNAs^[11,12]^. Mirtrons generate pre-miRNAs after splicing and trimming of tail residues in the nucleus, independently of Drosha, followed by further processing through the canonical biogenesis pathway^[13,14]^. tRNA-derived miRNAs can arise either from precursor tRNAs or from the 5’ or 3’ termini of mature tRNAs through mechanisms that are not yet completely understood. Current evidence, however, indicates that the synthesis of miRNAs derived from precursor tRNAs requires RNase Z, whereas the synthesis of miRNAs from mature tRNA depends on Dicer^[11,15]^.

Once RISC assembly is complete, mature miRNAs can bind to their target messenger mRNAs, thereby suppressing expression of the encoded protein. In plants, miRNA-mRNA interactions typically involve perfect or near-perfect complementarity, leading to the direct cleavage of the mRNA by the RNA interference machinery. In animals, binding is typically imperfect, usually involving the miRNA’s seed sequence binding to complimentary sites within the 3’ UTR of target mRNAs, and results in translational repression or mRNA degradation^[5,16]^. miRNAs can be stably detected in blood and other bodily fluids. They can be transported between tissues, individuals, and even species, including between pathogens and hosts, primarily via extracellular vesicles (EVs). EVs deliver miRNA cargo to recipient cells, where miRNAs can exert biological functions^[17,18]^. It is increasingly evident that miRNAs participate in nearly all physiological and pathological processes, including development, immune homeostasis, infection, immunity, and disease pathogenesis.

### Biogenesis of EVs

EVs comprise a diverse population of membrane-bound particles released into the extracellular environment via distinct, highly regulated biogenesis pathways. They are broadly categorized into two primary subtypes, exosomes and ectosomes. EVs are ubiquitously secreted by known all cell types across domains of life and are readily detectable in a spectrum of biofluids, including blood, urine, tears, and breast milk, where they serve as key mediators of intercellular communication^[19-26]^.

The distinction between these EV classes is fundamentally rooted in their site of origin. Ectosomes (100-1,000 nm), formerly referred to as microvesicles, arise through direct outward budding followed by fission from the plasma membrane. This process is driven by localized actomyosin cytoskeletal remodeling and loss of phospholipid asymmetry, specifically through scramblase-mediated translocation of phosphatidylserine to the outer leaflet^[27,28]^. Ectosome release can be divided into slow- and fast-release mechanisms. The slow-release mechanism can take several minutes and is characterized by increased intracellular calcium levels and subsequent membrane shedding. In contrast, the fast-release mechanism occurs within approximately one minute via vesicle extrusion through a pore in the plasma membrane^[29]^. Conversely, exosomes (30-150 nm) originate within the endosomal system. Their biogenesis begins during the maturation of early endosomes into late endosomes or multivesicular bodies (MVBs), which are characterized by the formation of intraluminal vesicles (ILVs)^[23,25]^. This inward invagination is primarily orchestrated by the Endosomal Sorting Complex Required for Transport (ESCRT) machinery, including ESCRT-0 through ESCRT-III, which facilitates the selection of ubiquitinated cargo and membrane scission. During this process, ubiquitinated cargo is recognized and clustered by ESCRT-0 through ESCRT-II. Subsequently, ESCRT-III and associated proteins, such as ALIX and VPS4, form ILVs by mediating membrane deformation and scission^[30-32]^. Complementary ESCRT-independent mechanisms, involving tetraspanins and ceramide-induced budding, contribute to ILV diversification^[28,33]^. Upon regulated MVB fusion with the plasma membrane, these ILVs are released into the extracellular space as exosomes.

The regulation of EV biogenesis involves a complex interplay among ESCRT components, lipid rafts, tetraspanins, and cytoskeletal dynamics. Regardless of their origin, EVs encapsulate a rich repertoire of bioactive molecules, including proteins, lipids, and various RNA and DNA species, that reflect the physiological or pathological state of the donor cell^[23,25,34]^. By facilitating the horizontal transfer of this molecular cargo to recipient cells, EVs serve as critical mediators of intercellular communication, immune modulation, and disease progression, particularly in the tumor microenvironment^[34]^.

In addition to exosomes and ectosomes, apoptotic bodies represent a third class of EVs. These vesicles range from 50 nm to 5 µm and are generated via membrane blebbing during programmed cell death^[35]^. Like other major EV subtypes, apoptotic bodies can encapsulate nucleic acids, proteins, and viruses^[36,37]^. While their heterogeneous sizes often lead to co-isolation with other EV subtypes, they are traditionally distinguished by their origin from nonviable cells and their rapid phagocytic clearance by macrophages. Nevertheless, emerging evidence suggests that apoptotic bodies derived from malignant cells harbor critical proteomic and genomic information, supporting their potential utility for specialized applications in cancer diagnosis and liquid biopsies^[38]^.

### EVs and their miRNA cargo

As described above, EVs are enriched in small functional RNAs^[39-41]^. Among their diverse cargo, EV-associated RNA species, including miRNAs, mRNAs, small nuclear RNAs (snRNAs), snoRNAs, tRNAs, and long non-coding RNAs (lncRNAs), have emerged as critical regulators of physiological and pathological processes^[40,42-48]^. Through selective packaging and protection against degradation, EVs enable the stable transfer of RNA to recipient cells in both local and distant environments^[41,49,50]^. The packaging of miRNAs into EVs is a highly regulated process that enables the secretion of specific miRNAs and facilitates cell-to-cell communication through the transfer of genetic material. RNA-binding proteins, such Y-box-binding protein 1 (YBX1), the SUMOylated form of the heterogeneous nuclear ribonucleoprotein A2/B1 (hnRNPA2B1), and major vault protein (MVP), play essential roles in binding and directing miRNAs into vesicles^[24,43,51-54]^. Additionally, RNA-binding proteins recognize sequence motifs within miRNAs, such as UGGA and GGAG *EXO-motifs*, thereby facilitating their packaging into EVs^[23,52,54]^. Intracellular signaling pathways, including the ceramide-dependent pathway, further regulate this process. Other factors, such as cellular stress and environmental cues, can influence the sorting of miRNAs into EVs^[24,34,52,55]^. Once packaged, miRNAs remain stable and protected from degradation, allowing them to travel through the extracellular environment and bloodstream before uptake by recipient cells, where they can influence gene expression and modulate biological processes^[24,34,44,55]^. Advances in RNA sequencing (RNA-seq) technologies and EV isolation methodologies have greatly improved our understanding of EV RNA profiles and their diagnostic and therapeutic potential^[56]^; however, the standardization of EV-RNA analyses and the elucidation of mechanisms governing selective RNA loading and functional transfer remain major unsolved challenges.

This review provides a comprehensive overview of EV-associated miRNAs by evaluating current methodologies for EV collection and isolation, RNA extraction, and miRNA quantification, while also addressing key pre-analytical and analytical challenges that affect data quality and reproducibility. This review also highlights ongoing standardization efforts and discusses the diagnostic potential of EV-derived miRNAs across diverse biofluids as biomarkers in modern molecular diagnostics.

## COLLECTION OF EVS FROM BIOFLUIDS

EVs can be isolated from a wide range of biofluids, providing a minimally invasive window into physiological and pathological processes^[25,47,55,57-72]^. Although each biofluid offers distinct advantages, EV isolation and downstream analyses are consistently influenced by pre-analytical and methodological variables, including sample collection, anticoagulant selection, processing time, storage conditions, pH, and mechanical handling, all of which require standardization to ensure reproducibility^[24,73-79]^. Moreover, residual biological activity may persist after sample collection, and cells present in biofluids can continue to release EVs and EV-associated miRNAs during the interval between collection and processing, even in the absence of hemolysis^[80,81]^. Such *ex vivo* alterations may reduce analytical accuracy by distorting EV composition and biomarker profiles^[82]^. While this phenomenon is best characterized in blood, similar handling-dependent changes have been reported in other biofluids, including urine and saliva, highlighting a broader challenge in EV-based diagnostics^[83-85]^. EV isolation typically involves initial low-speed centrifugation to remove cells and debris, followed by enrichment techniques such as ultracentrifugation (UC), size-exclusion chromatography (SEC), affinity capture, or precipitation^[61,70,77,86-90]^. The selected isolation methodology substantially influences EV purity, recovery, and downstream performance, particularly because of the frequent co-isolation of non-vesicular contaminants, including protein complexes and lipoproteins^[77,86]^. Encapsulated within the EV lipid bilayer, miRNAs exhibit extracellular stability, resistance to nuclease-mediated degradation, and reliable detectability in circulation. Together with their relative abundance and disease-associated expression patterns, these properties position EV-associated miRNAs as minimally invasive biomarkers for diagnostic and prognostic applications^[24,59,60,74,91-102]^. Moreover, additional EV-associated molecular cargoes, including DNA-based alterations such as mutations and methylation signatures, as well as proteomic and lipidomic profiles, represent complementary biomarker sources with potential clinical relevance^[103-128]^.

### Well-studied biofluids and prominent liquid biopsies

#### Blood

Blood is a highly informative biofluid for EV analysis, providing insights into systemic physiological and pathological processes. EV isolation from plasma or serum typically begins with low-speed centrifugation to remove the cells and platelets, followed by one or more enrichment methods.

#### Urine

Urine is a non-invasive biofluid that provides a readily accessible source of EVs for disease monitoring, particularly in renal, urogenital, and systemic diseases, owing to its detectable and disease-associated miRNA cargo.

#### Tissue and organ culture supernatants

Many studies have used homogeneous 2D cell cultures or biofluids as sources of EVs. However, these sources often fail to fully capture the complex EV signaling networks and molecular signatures generated by the diverse cell populations within tissue or organ microenvironments. Organoids, 3D multicellular spheroids, and tissue-derived EVs (Ti-EVs) have been used to isolate and study EVs *in vitro* and *ex vivo* across normal physiological and pathological states. These Ti-EVs are present in the interstitial spaces between cells and may reflect both local and systemic EV-mediated communication. They have been isolated from multiple organ sites, including the brain, heart, liver, adipose tissue, and skeletal muscle and have the potential to provide insights into intercellular signaling across complex tissues and mechanisms of disease progression^[129,130]^.

### Collection of organ-specific EVs from unique biofluids

Although biomarker research has focused on detecting and identifying disease-associated circulating multi-omic analytes, recent studies have demonstrated the diagnostic utility of human biofluids that originate from, drain, or directly contact diseased tissues or organs of interest^[131,132]^. For example, because of its non-specific symptoms and frequent late-stage diagnosis, lung cancer remains one of the leading causes of cancer-related mortality worldwide. Although low-dose computed tomography (LDCT) screening has been developed to improve diagnostic accuracy, its clinical adoption has remained low because of its cost and limited ability to distinguish benign from malignant nodules^[133,134]^. Numerous biomarker studies have evaluated the diagnostic potential of circulating lung tumor biomarkers; however, none have achieved the sensitivity required for early lung cancer detection^[135,136]^. Because the lung can be directly accessed from the external environment, biomarker studies have shifted toward the collection and multi-omic analysis of airway biofluids, which may improve detection and facilitate testing^[137]^. These biofluids include saliva, bronchoalveolar lavage fluid (BALF), and exhaled breath condensate (EBC). They show promise for detecting lung cancer and other critical lung diseases, such as chronic obstructive pulmonary disease (COPD), as they contain airway- and lung-associated EV populations that can be selectively purified and analyzed^[59,138]^.

#### Saliva

Saliva is a biological fluid produced by the salivary glands, and its composition can change in response to oral or systemic disease. High EV yields have been recovered from as little as 0.2 mL of saliva using flocculation via orbital acoustic trapping (FLOAT)^[139]^.

#### BALF

The current clinical evaluation of BALF involves assessing both cellular components (e.g., macrophages, lymphocytes, and epithelial cells) and non-cellular components (e.g., viruses, bacteria, and inorganic dust), which can provide important insights into infectious, immunological, and inflammatory processes in the lung^[140]^. It is well established that BALF is rich in functional EVs secreted by airway cells, immune cells, and bacteria^[141,142]^.

#### Exhaled breath

Exhaled breath has been shown to carry both volatile organic compounds (VOCs) representing gases exchanged between the circulation and the external environment, and non-volatile organic compounds (non-VOCs), including metabolites such as nitrite, urea, amino acids, and macromolecules, including proteins, RNA, and DNA that become aerosolized from the fluid lining the lower respiratory tract^[143-146]^. For non-VOC collection, academic research laboratories and commercial companies have designed various condensing devices, such as the RTube and TurboDECC^[147,148]^. Furthermore, recent studies have shown that 1-2 mL of EBC can be collected within 10 min^[117,149,150]^. It has also been demonstrated that cancers originating from other organs can be detected in exhaled breath, as shown in a recent study by Half *et al.*, who successfully detected breast, colorectal, and prostate cancers, in addition to lung cancers, with high sensitivity and specificity using trained canines^[151]^.

#### Breast milk

Breast milk EVs are rich in bioactive molecules, including proteins, lipids, and nucleic acids; among these cargoes, miRNAs are particularly important regulatory molecules implicated in neonatal development^[55,88,152,153]^.

#### Nipple aspirate fluid

Nipple aspirate fluid (NAF) is a mammary gland-derived biofluid, typically obtained via a minimally invasive procedure using a sterile syringe or similar device to extract fluid from lactiferous ducts^[65]^. It may be secreted spontaneously or collected after induction by mechanical or chemical stimulation, such as oxytocin administration^[65,154]^. The utility of NAF as a diagnostic and therapeutic tool in breast cancer biomarker discovery is hindered by the limited volume of fluid obtained and its variable opacity^[152,155,156]^.

#### Sweat

Sweat produced by eccrine and apocrine glands contributes to thermoregulation. It helps maintain hydration, supports electrolyte balance, and provides a layer of innate immune protection at the skin surface^[157-160]^.

#### Tears

Tear-derived EVs are increasingly recognized as informative components of ocular fluid. They can carry DNA and RNA and may serve as promising non-invasive biomarkers for cancer^[62,95,161]^.

#### Cerebrospinal fluid

Cerebrospinal fluid (CSF), produced by plasma filtration and secretion in the choroid plexus, serves as a protective cushion for the central nervous system and supports nutrient transport, waste clearance, and immune homeostasis. Disruptions in its tightly controlled composition are commonly observed in neurological disorders^[162-164]^.

#### Amniotic fluid and umbilical cord blood

**Figure 1:**
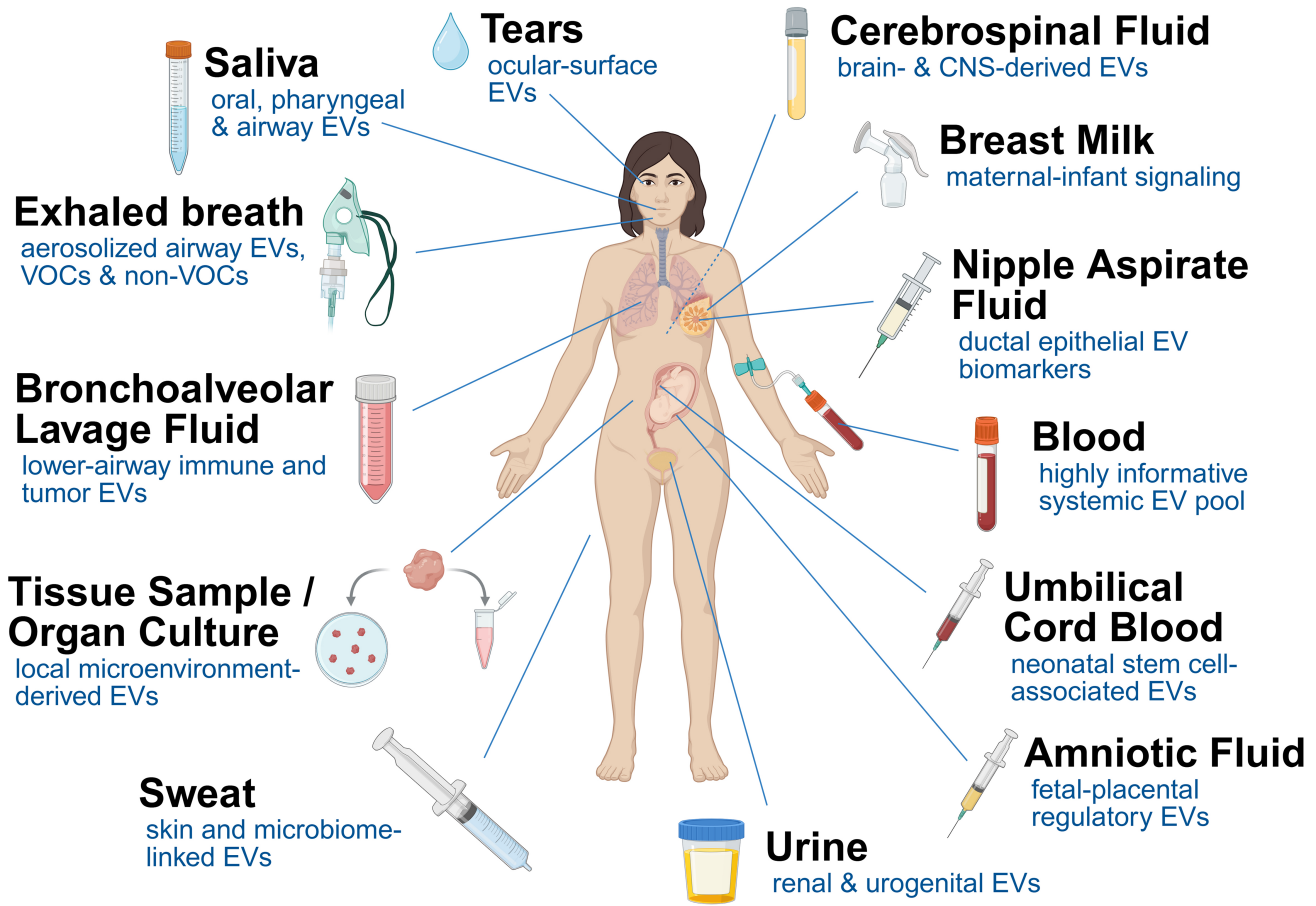
Schematic overview of different human biofluids as sources of EVs. Created wit BioRender. Grätz, C. (2026) https://BioRender.com/6gdw4pi. EVs: Extracellular vesicles; CNS: central nervous system; VOCs: volatile organic compounds.

Amniotic fluid- and umbilical cord blood-derived EVs, obtained either directly from these biological fluids or from mesenchymal stem cells (MSCs) derived from them, are emerging as valuable biological sources with considerable regenerative and diagnostic potential and relevance to therapeutic development and biomarker discovery^[58,63]^. Figure 1 summarizes the different body fluids mentioned above as sources of EV-associated miRNAs. Table 1 provides an overview of the biofluids and corresponding EV isolation techniques.

**Table 1:** Overview of EV isolation techniques for different human biofluids

**Biofluid**	**Common isolation methods**
Blood	low-speed centrifugation, UC, SEC, affinity-based purification, precipitation^[77]^
Urine	UC, filtration, precipitation, ultrafiltration combined with SEC, hydrostatic dialysis, acoustic trapping, immunocapture^[165]^
Tissue sample/organ culture	UC, density gradient^[166,167]^
Saliva	UC, co-precipitation, affinity-based purification, acoustic trapping^[86,139]^
BALF	UC, filtration^[168,169]^
Exhaled breath	UC^[117]^
Milk	UC, precipitation^[70,87,88,90]^
NAF	precipitation^[170]^
Sweat	UC, affinity-based purification, ultrafiltration^[96,103,171]^
Tears	UC, SEC^[62,95,161]^
CSF	UC, precipitation^[71,172,173]^
Amniotic fluid/umbilical cord blood	UC, ultrafiltration^[97,110]^

EV: Extracellular vesicle; UC: ultracentrifugation; SEC: size-exclusion chromatography; BALF: bronchoalveolar lavage fluid; NAF: nipple aspirate fluid; CSF: cerebrospinal fluid.

### EV-miRNA biomarkers across biofluids and disease applications

Several studies have directly compared the diagnostic performance of EV-associated miRNAs across different biofluids, demonstrating that biofluid selection can influence sensitivity, specificity, and clinical utility in a cancer-type dependent manner. For example, a recent meta-analysis in oral squamous cell carcinoma quantified the pooled sensitivities and specificities of circulating miRNAs detected in saliva, plasma, and serum. Although broadly similar diagnostic accuracies were observed across these fluids, with some variation in area under the curve (AUC) values and no statistically significant overall matrix effect, serum often exhibited numerically higher diagnostic performance than saliva or plasma when analyzed individually^[174]^. In prostate cancer, EV-associated miRNAs isolated from plasma have shown superior diagnostic and prognostic discrimination compared with cell-free miRNAs, with robust receiver-operating characteristic (ROC) AUC values and clinicopathological correlations, underscoring the value of EV enrichment in blood-based liquid biopsy approaches^[175]^. In lung cancer, a recent mini-review of EV-miRNAs in serum/plasma and airway fluids, such as BALF, reported distinct profiles that may reflect proximity to the tumor and could inform early detection and/or disease progression. However, direct head-to-head quantitative comparisons of sensitivity and specificity remain limited^[176]^. Similarly, CSF EV-miRNAs such as miR-21 in glioblastoma have been shown to discriminate central nervous system tumors with high sensitivity and specificity, in a manner distinct from systemic blood-based measurements, underscoring that biofluid origin influences biomarker performance and that tumor-proximal fluids may offer enhanced detection in certain contexts^[177]^. Taken together, these studies illustrate that comparative analyses across liquid biopsy routes are emerging and that the choice of biofluid may differentially affect diagnostic and prognostic outcomes depending on the tumor type, anatomical site, and disease biology.

## PURIFICATION OF EVS

**Figure 2:**
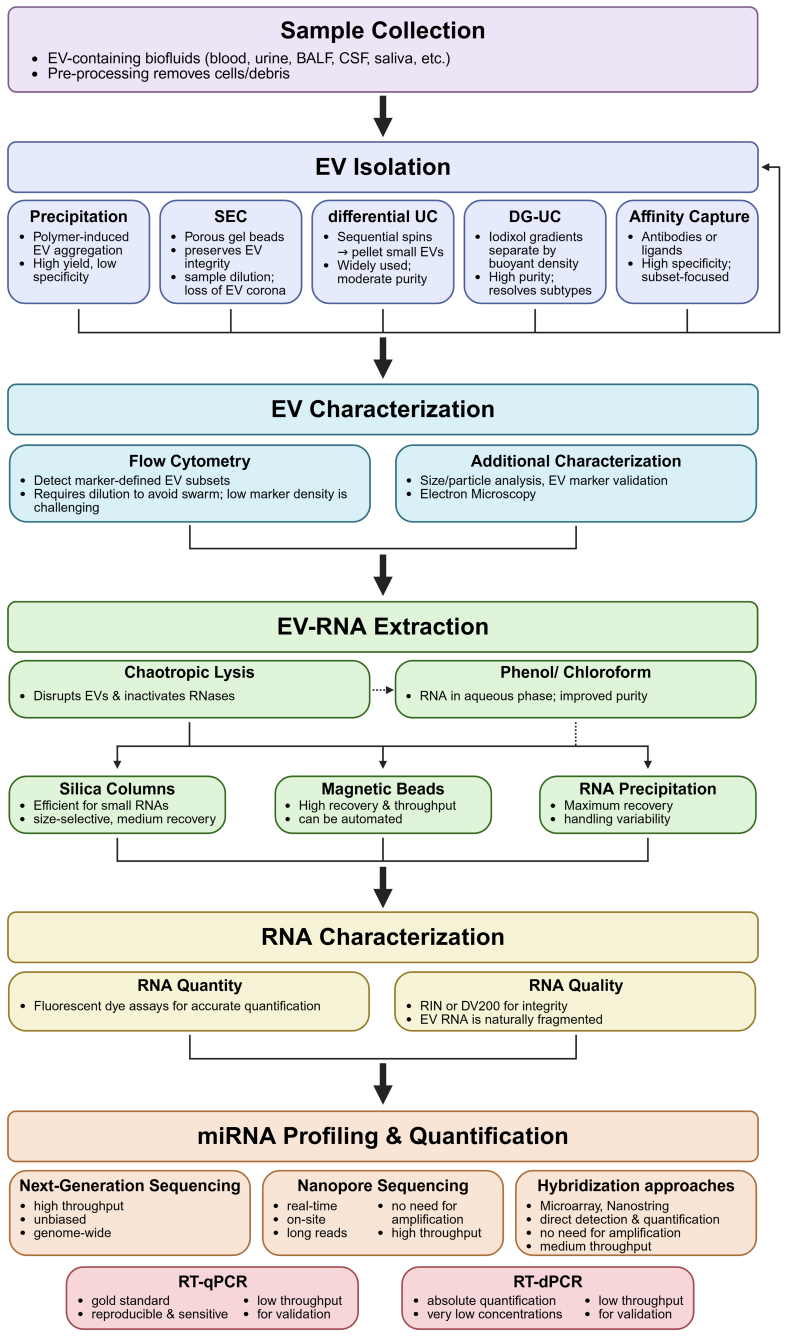
Flowchart of EV-miRNA analysis and quantification workflow. Created in BioRender. Grätz, C. (2026) https://BioRender.com/c2ir709. EV: Extracellular vesicle; miRNA: microRNA; BALF: bronchoalveolar lavage fluid; CSF: cerebrospinal fluid; SEC: size exclusion chromatography; UC, ultracentrifugation; DG-UC: density-gradient UC; RIN: RNA integrity number; DV200: percentage of RNA fragments of > 200 nt; RT-qPCR: reverse transcription-quantitative polymerase chain reaction; RT-dPCR: reverse transcription-digital polymerase chain reaction.

EVs (exosomes, ectosomes, and apoptotic bodies), as well as more recently described supermeres^[178]^, can be effectively isolated from various biofluids and supernatant from conditioned tissue culture media using differential ultracentrifugation (dUC), a widely adopted and standardized technique. This process involves a series of centrifugation steps with increasing centrifugal forces to sequentially remove cells, debris, and larger vesicles, culminating in the pelleting of small EVs^[77,179]^. dUC is compatible with various sample types, including plasma, serum, urine, and tissue culture supernatants, and remains a cornerstone of EV research despite limitations such as the potential co-purification of protein aggregates or lipoproteins^[180-182]^. Accordingly, dUC is frequently combined with complementary purification approaches, such as precipitation-based protocols, column-based purification (e.g., SEC), or density gradient ultracentrifugation (DGUC), which enables the resolution of EVs into distinct subpopulations according to their biophysical properties, including size, morphology, mass, and density. An overview of the entire EV-miRNA analysis and quantification workflow, from EV purification to miRNA characterization, is presented in the flowchart shown in Figure 2.

### Precipitation

Precipitation-based methods for EV isolation rely on organic solvents, sodium acetate, and polymers, such as polyethylene glycol (PEG), to reduce EV solubility and facilitate their precipitation^[141]^. Commercial kits for polymer precipitation include ExoQuick (System Biosciences), Total Exosome Isolation Reagent (Invitrogen), ExoPrep (HansaBioMed), Exosome Purification Kit (Norgen Biotek), exoEasy (Qiagen), and miRCURY Exosome Isolation Kit (Exiqon)^[90]^. Polymer-based EV precipitation methods involve the addition of a precipitation reagent to a biological sample, such as conditioned cell culture media, serum, or urine, followed by incubation to promote polymer-mediated aggregation and capture of EVs. The resulting EV-polymer complexes are subsequently recovered by low-speed centrifugation, resuspended, and processed for downstream analyses. Because these methods are technically straightforward and do not require specialized equipment, they remain accessible and widely used for routine EV isolation procedures. However, because precipitation-based methods frequently co-precipitate non-vesicular contaminants, including soluble protein aggregates, lipoproteins, and other extracellular particles, they necessitate additional purification strategies, such as DGUC, for downstream applications. Despite these limitations, precipitation-based techniques remain widely used because of their ease of use, scalability, and ability to process large sample volumes. Notably, several studies have reported that precipitation yields higher RNA and miRNA read counts than more stringent, higher-purity EV isolation methods^[183-185]^.

### SEC

SEC is a column-based EV purification technique that separates particles according to hydrodynamic size. In this approach, the sample is applied to a column containing porous gel beads of a defined pore size. Smaller particles enter the bead pores and traverse a longer path through the column, whereas larger particles are excluded from the pores and pass through the spaces between the beads, resulting in earlier elution of larger particles relative to smaller particles^[78,186,187]^. Column pore size, commonly approximately 75, 35, or 20 nm, strongly influences EV recovery, resolution, and purity, and can be selected based on the EV subtype of interest. In general, smaller pore sizes improve the resolution and purity of small EV preparations by reducing co-elution with soluble, non-vesicular proteins^[188]^. Because SEC does not require high centrifugal forces, chemical modification, or irreversible binding interactions, it is generally regarded as a gentle purification strategy that preserves EV integrity and biological activity, making it well-suited for downstream functional analyses^[189]^. However, SEC has several important limitations. First, the process inherently dilutes the sample, as the EVs are usually distributed across multiple eluted fractions, with each fraction often approximating the starting sample volume^[190,191]^. Recent data suggest that SEC may partially strip EVs of their functional protein corona, which could alter EV function in downstream applications^[192,193]^. Therefore, for studies requiring highly concentrated EV preparations or preservation of functional protein corona, SEC may be suboptimal unless combined with complementary strategies^[194]^.

### Density-gradient purification

Crude EVs isolated via dUC can be further resolved into distinct populations based on physical properties, such as size, shape, mass, and/or buoyant density, using DGUC^[74,195]^. DGUC is generally classified as one of the following: isopycnic, equilibrium zonal, and rate-zonal. Each method utilizes a density gradient generated using a high-density medium, such as iodixanol, in combination with an ultracentrifugal force to achieve high-resolution EV fractionation. In an isopycnic DGUC, a crude EV sample is layered onto or within a discontinuous gradient composed of a medium with progressively increasing densities^[196]^. Centrifugation is then performed until a stable linear density gradient is formed, separating the EV sample into discrete subpopulations based on their buoyant density^[196]^. Similarly, equilibrium zonal DGUC separates EVs primarily according to buoyant density; however, centrifugation is continued until particles reach their characteristic buoyant density within the gradient^[197]^. In contrast, rate-zonal DGUC uses a preformed linear density gradient to separate EVs by sedimentation behavior, thereby enabling fractionation based on particle size, mass, and sedimentation coefficient^[198]^.

### Affinity-based purification

Affinity-based EV purification utilizes selective interactions between components of theEV’s surface and immobilized ligands to enrich for EV populations from biofluids. Ishida *et al*. demonstrated that chromatographic purification of EVs from conditioned cell culture media and CSF could be achieved using lysine-rich peptides with affinity for phospholipid membranes^[199-201]^. Specifically, peptides containing 8 or 16 lysine residues were shown to bind to phospholipid membranes and enable the capture of EVs using affinity bead-based column chromatography^[199,200]^. A key advantage of this approach is its compatibility with mild elution conditions, allowing EVs to retain their spherical shape^[199]^. However, initial studies were performed using relatively small input volumes (≤ 500 µL), which limit the application’s scalability for large-scale EV isolation. To address this, companies such as Siliconbio, Inc. (Hiroshima, Japan) developed ExoPUA, a cellulose-resin column with immobilized peptides containing 25-35 lysine residues. Using ExoPUA, Masaki *et al*. showed that this approach increased column capacity and selectivity for EV-associated targets, thereby improving the overall efficiency of the separation process to a level comparable to that of DGUC^[200]^.

#### Immunopurification

Because the EV surface membrane contains both canonical EV-associated markers (i.e., tetraspanins CD9, CD63, and CD81^[202]^) and cell type- or context-specific surface proteins, including receptors and transmembrane proteins^[203]^, that mediate interactions with recipient cells and extracellular ligands, antibody-based isolation techniques have been developed to enrich EVs from diverse biofluids^[204-206]^. Immunopurification strategies generally use either individual or combined biotinylated anti-tetraspanin antibodies immobilized on streptavidin-coated magnetic beads^[207]^. However, a recent study by Mitchell *et al.* demonstrated that magnetic beads may also co-isolate unwanted contaminants that can interfere with downstream transcriptomic analyses, in part because of their affinity for nucleic acids^[208]^.

#### Galectin-coated magnetic beads: EXÖBeads

EXÖBead isolation uses Galectin-coated magnetic beads to capture EVs while minimizing lipoprotein contamination and preserving vesicle integrity. This approach offers a streamlined workflow and enables recovery of intact EVs using a lactose-containing elution buffer, as confirmed by cryo-electron microscopy (cryo-EM) and nanoparticle tracking analysis (NTA). EXÖBeads were first applied in head and neck squamous cell carcinoma (HNSCC) to isolate high-purity immunosuppressive plasma-derived EVs^[209]^. In patients with relapsed HNSCC, Collectin Subfamily Member 10 (COLEC10) was identified as a recurrence-associated EV protein implicated in complement activation and TNF-like signaling pathways^[210]^. EXÖBeads have also been successfully applied in amyotrophic lateral sclerosis (ALS)^[211]^. Although this approach is promising, EXÖBeads remain an in-house methodology, are not yet readily accessible, and require more extensive, large-scale benchmarking.

#### Other affinity-based purification methods

To target common EV surface features, recent affinity-based strategies have employed ligands other than antibodies, such as aptamers^[212]^ and Tim-4 protein^[213]^, which bind phosphatidylserine exposed on the EV surface, thereby enabling EV immobilization on magnetic beads^[214]^ or capture on microtiter plates for ELISA-based applications^[215]^. Although antibody- and affinity-based approaches can efficiently enrich EVs from a given biofluid, methods directed against broadly expressed EV markers generally yield bulk EV populations rather than cell- or tissue-specific subsets^[195]^. Accordingly, antibody-based isolation platforms have increasingly been engineered to capture EVs using cell-specific surface markers^[208,216,217]^.

#### Applications for affinity-based methods

These approaches enable the identification of cell-specific, EV-associated circulating biomarkers in multiple human cancers^[208]^ and permit the evaluation of their biological functions *in vitro*, provided that the isolated EVs can be efficiently released from the capture matrix without compromising vesicle integrity or activity^[218,219]^. However, EV isolation using a single antibody may be insufficient for cell-type-specific enrichment because many surface proteins are shared across multiple tissues and cell lineages. Therefore, antibody-based technologies have been developed to define the EV surfaceome, including the Proximity Extension Assay (PEA), a quantitative polymerase chain reaction (qPCR)-based assay that uses antibody pairs conjugated to unique oligonucleotides, enabling highly sensitive detection of EV surface proteins when paired probes are brought into proximity^[220,221]^. Substantial effort has also been devoted to adapting existing fluorescent antibody-based labeling methodologies to quantify and potentially separate individual EVs for downstream molecular analyses by flow cytometry (FCM)^[214,222]^. However, the small size of EVs is a hurdle in the development of FCM-based EV analyses. Consequently, the next generation of antibody-based EV isolation assays has focused on developing microfluidic platforms^[223,224]^. These technologies aim to integrate selective EV capture, quantification, surfaceome profiling, and transcriptomic cargo analysis into powerful diagnostic assays^[224,225]^.

### FCM

To date, most FCMs have been optimized for cellular analysis, with detection of particles ranging from 8 to 15 µm in size^[226]^. For cell-based applications, fluorescent signals generated by antibody-mediated recognition of cellular epitopes, together with and light-scattering signals, can generally be distinguished from background noise^[226]^. However, when analyzing nanoparticles such as EVs, which typically range from 30 to 500 nm in diameter, background noise can substantially hinder the discrimination between true EV-associated events and noise, making signal-to-background resolution a critical determinant of data interpretation. Indeed, EV-FCM often generates positive events that overlap with background signals arising from sheath fluid, buffer components, antibody aggregates, unbound fluorophores, protein complexes, lipoproteins, and other particulate contaminants. Consequently, sensitive, reproducible, and quantitative EV detection, immunophenotyping, and molecular characterization remain technically challenging^[227]^. Moreover, the lack of standardized EV isolation and processing methods contributes to substantial heterogeneity in reported EV size distribution, concentration, and compositional properties. Given the difficulties associated with interpreting and comparing data across diverse EV preparations and FCM platforms, the International Society for Extracellular Vesicles (ISEV), International Society for the Advancement of Cytometry (ISAC), and International Society on Thrombosis and Hemostasis (ISTH) jointly established the EV Flow Cytometry Working Group (www.evflowcytometry.org).

#### Visible light scattering properties of EVs

As EVs pass through the FCM laser beam, they scatter light less efficiently and with different optical behavior than larger cells or solid, compositionally homogeneous calibration particles such as polystyrene beads^[228]^. This difference is largely attributable to the small diameter and low refractive index of EVs, which are influenced by their macromolecular composition, organization (i.e., membrane *vs.* lumen), and overall size. Therefore, particle refractive index, vesicle heterogeneity, calibration strategy, and instrument sensitivity must be carefully considered when interpreting EV light-scattering measurements by FCM.

#### EV stability

The sheath flow, maintained under pressure, establishes a defined path for the EV-containing sample to traverse through the laser beam. To preserve EV stability during analysis, both the sheath and sample fluids are maintained under isotonic conditions, commonly 1× PBS^[227,229]^. However, salts, protein aggregates, residual debris, and microparticulate contaminants present in biofluids or buffers may contribute to fluidic instability and clogging, thereby increasing background signal and reducing overall flow rates^[230,231]^. Consequently, EV-FCM instruments often incorporate fluidic filter systems to remove impurities and particulate contaminants, and some newer FCM platforms require ultrapure water-based fluidics.

#### EV detection and quantification

Because EVs vary widely in abundance, size, and molecular composition, the number of detectable fluorescently labeled antibodies bound to the EV membrane can vary tremendously, depending on surface antigen density, epitope accessibility, fluorophore brightness, antibody affinity, and steric constraints. Consequently, EVs expressing low levels of surface biomarkers may fail to generate a signal sufficient to distinguish them from background noise. Indirect immunostaining using primary and secondary antibodies can further exacerbate background fluorescence, as secondary antibodies may engage in non-specific binding interactions, and the additional incubation and washing steps inherent to indirect staining can amplify reagent-derived background and non-specific signal^[232,233]^. To address these limitations, companies developing EV-FCM instrumentation have focused on engineering highly sensitive fluorescent probes and membrane-associated dyes, such as MemGlow or CellMask, to improve the detection of small EVs^[234]^. In parallel, the development of Fc-free affinity reagents, including antibody fragments and engineered binding scaffolds such as monobodies, may reduce non-specific signal arising from Fc-mediated interactions, antibody aggregation, and contaminating immune complexes. Collectively, these advances reflect significant efforts to minimize background fluorescence from antibodies and reagents. In addition, appropriate control experiments, including antibody and buffer pre-filtration, detergent lysis controls, unstained controls, isotype controls, fluorescence-minus-one (FMO) controls, and serial dilution analyses, are essential for identifying noise and non-specific events, thereby improving gating strategies and the accurate detection of EV-derived signals^[235]^. Furthermore, automated software tools, such as FlowClean and FlowAI, have been developed to identify and remove flow-rate fluctuations, signal instability, and artifactual fluorescence anomalies in FCM datasets, enhancing data quality and reproducibility of analysis^[236]^.

#### FCMs for EV analysis

A range of FCM platforms has been evaluated for EV analysis, including conventional instruments retrofitted to detect nanoscale particles, despite not being originally designed for this purpose. More recently, high-sensitivity FCMs have been specifically engineered to reduce background noise and improve EV detection limits. Several platforms have demonstrated promising potential for EV analysis, including the Flow Nanoanalyzer from NanoFCM^[237]^, Beckman Coulter CytoFlex Nano^[238]^, and Cytek Aurora^[239]^ systems. In addition to optical FCM, resistive pulse sensing-based particle analyzers, which detect EVs as they pass through an electrically biased nanopore, provide an alternative approach for EV sizing and quantification, including assessment of surface markers. One such platform is the Spectradyne ARC instrument, although its utility for multiparametric phenotyping is limited by the relatively small number of available fluorescence channels.

### FCM-based EV sorting

In addition to EV characterization, FCM can be used to sort EVs based on fluorescently labeled markers. Groot Kormelink *et al*. improved EV sorting using high-resolution FCM by carefully controlling the swarm effect, in which multiple particles simultaneously pass through the interrogation point and distort signal intensity and event counting. Through serial dilutions, they established linear event rates and reliable single-particle detection. Post-sort analysis and immunoblot analysis confirmed the high-purity separation of CD9^+^ and CD63^+^ EV subpopulations^[240]^. Similarly, Morales-Kastresana *et al.* introduced a NanoFACS approach that uses fluorescence-triggered FCM to sort individual EVs and viral particles. By applying serial dilutions and specific fluorescent labeling, they minimized the swarm effect and achieved gentle, high-purity sorting with confirmed vesicle recovery and preservation of EV integrity^[241]^. Pieragostino *et al.* used optimized polychromatic flow cytometry (PFC) to isolate highly pure EV populations from the CSF and tears of patients with multiple sclerosis (MS) and healthy controls. Their findings identified a molecular link between CSF- and tear-derived EVs, which contain a distinct network-structured cargo of pathological proteins. This cargo included activated upstream regulators such as TGFB1, ANGPT2, HIF1A, and IL-4, as well as proteins involved in angiogenesis and immune responses, suggesting that EV cargo may reflect the central nervous system’s immune status and disease-associated signaling programs^[242]^. Although these studies expand the technical boundaries of EV sorting, the approaches remain highly dependent on specialized instrumentation, rigorous calibration, optimized staining conditions, and finely tuned acquisition settings, which currently limit their reproducibility and broader implementation across laboratories.

## RNA ISOLATION FROM EVS

### Comparison of EV-RNA isolation methodology

Given that EVs contain intact small RNAs, predominantly miRNAs, as well as fragmented mRNA and lncRNA species, manufacturers have developed or adapted filter-based column kits to efficiently isolate EV-associated RNA cargo. Some kits enable the direct isolation of cell-free RNA (cfRNA) from various biofluids, whereas others require prior EV concentration or enrichment. In many cases, EV enrichment and RNA isolation are integrated into a single workflow, although most products are optimized for pre-concentrated EV samples.

RNA isolation typically begins with incubation in guanidinium thiocyanate, a chaotropic agent that disrupts EV membranes and inactivates RNases^[243]^. Following lysis, samples are often separated into phases by adding chloroform to the phenol-containing lysis buffer, followed by centrifugation. This step removes most contaminants, including proteins, lipids, and DNA. The aqueous phase is then recovered, mixed with alcohol, and applied to a silica-based filter column. Alternative approaches bind RNA to magnetic beads, which may improve RNA recovery relative to conventional column-based methods in some workflows^[244]^, or employ lysis chemistries that avoid phase separation altogether. In addition to silica- and bead-based methods, RNA can be isolated through precipitation and centrifugation, which generally increase total RNA yield, but are more sensitive to handling variability. Since approximately 30% of RNA is typically lost during silica column-based purification, and the loss is even higher for smaller RNA fragments, non-column-based isolation strategies may be advantageous for EV-associated RNA, which is often fragmented and present at very low abundance^[245]^.

Once RNA is bound to silica matrices or bead surfaces or recovered by precipitation, multiple wash steps are required to remove potential contaminants; however, these steps may also reduce RNA yield^[246]^. Carrier RNA can be added to improve recovery. However, this is typically unsuitable for biomarker discovery studies because carrier RNA cannot be removed prior to sequencing and may confound downstream library composition, normalization, and quantification. Instead, spike-in RNAs of known concentrations are commonly used to monitor isolation efficiency and sample-to-sample losses^[247]^. For silica column-based protocols, manufacturers such as Qiagen often offer options for the selective recovery of small RNA (< 200 nt), long RNA (> 200 nt), or both in a single eluent.

Importantly, next-generation sequencing (NGS) and targeted qPCR studies have also analyzed intact or partially degraded longer EV-associated RNA, including mRNAs and lncRNAs^[248,249]^, using similar chaotropic lysis and silica-based purification approaches. Various commercial kits (e.g., Qiagen, Norgen, Exiqon, or Macherey-Nagel) have been widely used in EV RNA workflows, and comparative studies have documented the performance of Qiagen RNeasy columns and similar silica-based protocols for extracting EV-associated RNA of sufficient quality for downstream molecular analysis^[250,251]^. For example, the Exiqon miRCURY RNA Isolation Kit has been used to extract total RNA from EVs for deep sequencing and profiling, whereas comparative miRNA studies have evaluated Macherey-Nagel NucleoSpin miRNA and Qiagen miRNeasy/RNeasy kits for exosome-associated RNA isolation, demonstrating their utility in EV-RNA workflows^[183,252,253]^. Because mRNAs and lncRNAs are longer than small non-coding RNAs, it is essential to choose a kit that supports consistent recovery across a broad RNA size range rather than selectively enriching small RNAs, as different RNA isolation protocols can introduce size-dependent biases and affect the recovery of long transcripts, such as mRNAs and lncRNAs^[254,255]^.

### Assessment of EV-RNA quantity, integrity, and miRNA stability

The assessment of the quantity and quality of isolated EV-RNA is crucial, as RNA yield, purity, and fragmentation can vary substantially among samples and directly influence downstream applications. Generally, RNA isolated from liquid biopsy EV samples is at least partially fragmented^[256,257]^. Several methods are available for this purpose; however, not all are suited for the distinct molecular characteristics of EV-associated RNA. UV-Vis spectroscopy, including optical density (OD) 260/280 and OD 260/230 ratios, can confirm the absence of contaminating solvents or proteins. However, its detection limit is usually too low to accurately determine EV-RNA concentration^[258-260]^. Methods that utilize fluorescent dyes, such as fluorometry and capillary gel electrophoresis, are more suitable for EV-RNA concentration measurements^[94]^. These methods, such as the Agilent Bioanalyzer 2100 and TapeStation, also assess the quality of the isolated RNA and report numerical values calculated from ribosomal RNA (rRNA) integrity or RNA fragment-size, such as the RNA integrity number (RIN) from the Bioanalyzer, or the percentage of RNA molecules > 200 nt (DV200) from the TapeStation. For EV samples, DV200 is generally more informative than RIN, as EV-associated RNA contains relatively low levels of intact rRNA^[261,262]^.

## QUANTIFICATION OF EV-miRNAs

### NGS

#### Small RNA-seq

Small RNA-seq is a powerful high-throughput technology used to comprehensively profile diverse small RNA species, including miRNAs and other regulatory non-coding RNAs, enabling unbiased, genome-wide quantification of small RNA populations^[40,56]^. Library preparation protocols for small RNA-seq differ from those for full-length RNA-seq in several critical respects. Because miRNAs are relatively low in abundance compared to longer RNA species, a gel- or bead-based size selection step is necessary after full-length libraries are generated. This size-selection step also reduces the need for further depletion of unwanted RNA species, such as rRNA, is often unnecessary. Size selection removes library molecules with inserts outside of the desired size range, as well as adapter dimers, resulting in a library with relatively uniform amplicon lengths. Furthermore, random hexamer priming, which is typically used for total RNA-seq, could introduce sequence-dependent bias and inefficient priming due to the short RNA length. Therefore, reverse transcription primers are usually designed to bind to adapter sequences that are ligated directly to RNA molecules^[263]^.

Illumina platforms are typically used for small RNA-seq experiments, as they remain the dominant short-read NGS platform. They employ bridge amplification technology, meaning that the library molecules bind to the flow cell at both ends via adapter sequences. The main limitation of Illumina technology, short-read lengths of < 300 bp, is not relevant to small RNA-seq experiments with total library lengths of only ~150 bp. However, because the bridge amplification approach relies on PCR during both library preparation and sequencing, it is prone to PCR bias; therefore, the use of unique molecular identifiers (UMIs) is recommended^[264]^.

Recent improvements in small RNA library preparation protocols and short-read-optimized bioinformatics pipelines have markedly increased the sensitivity and specificity of small RNA detection, even in low-input or challenging samples, such as biofluids and EVs^[45,265,266]^. In parallel, the development of improved reference databases has enabled the more accurate annotation of known transcripts and the detection of previously unannotated transcripts. These efforts have contributed to the creation of an extracellular RNA (exRNA) atlas that catalogs exRNA cargo types and their carriers in various human biofluids^[45]^. As EV-associated miRNAs continue to show strong promise as minimally invasive biomarkers, numerous recent EV RNA studies have focused specifically on miRNA profiling^[42,267]^. Despite these advances, technical biases introduced during library preparation and data normalization remain important sources of variability that must be considered when interpreting and comparing small RNA-seq datasets^[94]^.

#### Long-read sequencing

In recent years, long-read sequencing techniques, such as nanopore-based and Pacific Biosciences (PacBio) single-molecule real-time (SMRT) sequencing, have emerged as third-generation sequencing technologies^[268]^ techniques. These technologies enable single-molecule sequencing of long RNA or complementary DNA (cDNA) molecules allowing isoform-resolved transcriptome analysis.

However, owing to the reduced sequencing accuracy for RNAs < 300 nt and high sequencing errors near the ends of RNA molecules, nanopore technology is usually not the first choice for miRNA-seq. Nevertheless, recent advances have enabled the direct sequencing of full-length miRNA molecules and suggest that nanopore sequencing may eventually provide a feasible complementary approach for small RNA-seq^[269-271]^.

### Hybridization-based quantification

Despite the emergence of high-throughput NGS technologies, microarrays remain a cost-effective platform for RNA quantification, particularly for targeted analyses and translational or clinical applications^[272,273]^. Microarrays measure RNA abundance by hybridizing fluorescently labeled target RNA or cDNA to complementary oligonucleotide probes immobilized on a solid surface, with fluorescence intensity is proportional to transcript abundance^[274]^. Various microarray formats have been developed to target specific RNA populations, including oligonucleotide arrays, cDNA arrays, and specialized platforms designed for small and other non-coding RNAs^[41]^. Microarray profiling has made important contributions to biomarker discovery and disease classification^[275]^. However, key limitations - including cross-hybridization, restricted dynamic range, reduced sensitivity for low-abundance transcripts, and dependence on predefined probe content and prior sequence knowledge - sould be carefully considered^[276]^. Accordingly, RNA-seq has largely replaced microarray analysis for biomarker discovery.

As another hybridization-based platform, NanoString nCounter technology can directly detect and quantify transcripts without amplification, thereby facilitating the profiling of small RNAs^[272]^. NanoString assays utilize pairs of sequence-specific capture and reporter probes, in which reporter probes carrying target-specific fluorescent barcodes. This enables the quantification of individual RNA molecules through direct digital counting using single-molecule imaging^[277]^. Although NanoString is less suitable for unbiased transcript, it remains a valuable tool for targeted transcriptomic analyses, particularly in low-input samples.

### Reverse transcription qPCR and digital PCR

For decades, qPCR has been considered the gold standard for the reproducible and sensitive detection and quantification of gene expression^[278]^. Reverse transcription-qPCR (RT-qPCR) has supported the discovery of novel transcripts, the validation of RNA-seq data, and the reproducible detection of low-abundance transcripts^[40]^. In the context of EV-associated RNA, RT-qPCR is particularly important because EV-associated RNA cargo is often present in limited quantities, fragmented, and enriched for short RNA species^[44,272]^. However, normalization remains a critical analytical challenge and requires the careful validation of endogenous reference targets, exogenous spike-in controls, global mean approaches, or other context-appropriate strategies to ensure reproducibility across samples. Compared with high-throughput sequencing technologies, qPCR has substantially lower multiplexing capacity. Nevertheless, because of its rapid turnaround time, cost-effectiveness, and reproducibility, qPCR remains a cornerstone of molecular biology in both research and clinical settings. The advantages, limitations, and potential pitfalls of RT-qPCR for molecular biomarker discovery have been extensively reviewed previously^[279]^.

Digital PCR (dPCR) has gained considerable attention for detecting low-copy-number EV-RNA targets^[280]^. In dPCR applications, the reaction mix is partitioned into nanoliter-sized reaction partitions, such as wells or droplets, with individual PCR reactions performed in each partition. Partitions containing at least one copy of the template RNA or cDNA generate a fluorescent signal fluorescence detection. This enables absolute copy number quantification without a standard curve, even at very low concentrations^[281]^.

While NGS applications are typically the preferred method for unbiased biomarker discovery, RT-qPCR and RT-dPCR are generally used to validate biomarker candidates. Thus, these methods play complementary roles in EV-miRNA biomarker workflows.

## STANDARDIZATION IN EV-miRNA BIOMARKER STUDIES

### Methodological variability and its impact on EV research

The purification of EVs from heterogeneous biological sources and the characterization of their complex cargoes remain major technical and analytical challenges in the EV field. As described in Section “PURIFICATION OF EVS”, numerous methods have been proposed, developed, and utilized for the concentration and purification of EVs, including dUC, SEC, affinity-based methods, and precipitation-based techniques^[77,282,283]^. Characterization of the isolated EVs is usually performed using techniques such as NTA, dynamic light scattering (DLS), transmission electron microscopy (TEM) or cryo-EM, and FCM or immunoblotting for canonical EV-associated surface markers, including CD9, CD63, and CD81^[227,284-287]^. However, the choice of isolation and characterization methods is often complex and depends heavily on the biological source, sample volume, available instrumentation, and study objectives.

A central challenge in EV research is the trade-off between the yield, purity, and functional integrity. For instance, EVs overlap in size and density with non-vesicular structures commonly present in biofluids, such as lipoproteins and protein aggregates, which can be co-isolated when using size- or density-based approaches^[78,282]^. At present, no single concentration or purification method consistently achieves both high EV yield and purity across all sample types. Moreover, the recently proposed EV-associated protein corona may represent a biologically functional component rather than a simple contaminant; depending on the purification method, this corona may be co-purified, depleted, or misinterpreted as non-vesicular contamination potentially altering apparent EV composition and function^[192,193,288]^. These considerations highlight that purity, yield, and functional integrity must be carefully balanced rather than optimized in isolation.

Additionally, a substantial fraction of the EV population is smaller than 100 nm, which may be below the detection limit of some widely used instruments, such as conventional FCM platforms and certain NTA systems. Together, these limitations introduce significant methodological variability and potential bias into EV studies. These technical limitations, together with EV-specific challenges described in the “Minimal Information for Studies of Extracellular Vesicles” (MISEV), should be considered to ensure reproducibility. In the following sections, the most important guidelines and standardized frameworks are discussed in the context of EV-miRNA biomarker development.

### Standardization frameworks: MISEV and MIQE

To address methodological inconsistencies and support reproducible EV research, ISEV published its MISEV guidelines for the first time in 2014 (MISEV2014^[289]^ later updated in 2018 (MISEV2018)^[283]^ and most recently in 2023 (MISEV2023)^[78]^. These position papers aim to standardize EV research in terms of nomenclature, sample procurement, storage, and processing, and to outline the strengths and limitations of EV isolation and characterization methods across biological sources^[78]^. To further encourage reporting and documentation, the EV-TRACK database, launched in 2017, allows researchers to record the technical parameters of their published EV studies^[290]^.

Although MISEV is widely accepted within the EV research community, most EV-related papers published between 2015 and 2020 did not cite the guidelines. This observation is important because articles citing the MISEV guidelines also tended to use more EV characterization methods and markers than those that did not cite the guidelines^[291]^. Furthermore, a recent systematic review by Poupardin *et al*. demonstrated that different EV isolation methods correlate with distinct EV functions and cargoes, highlighting the risk of bias introduced by seemingly minor methodological decisions^[292]^.

Complementary to MISEV, reproducible downstream RNA analysis requires adherence to the Minimum Information for Publication of Quantitative Real-Time PCR Experiments (MIQE) guidelines. They were first established by Bustin *et al*. in 2009^[293]^ to increase the reproducibility and transparency of RT-qPCR experiments. Before their publication, researchers lacked consensus on how to design and conduct qPCR experiments, contributing to substantial variability between laboratories^[294]^. The original MIQE guidelines propose a standardized nomenclature for vital terms used in RT-qPCR and suggest best practices for all aspects of the experiment, from sample acquisition through quality control of the isolated nucleic acids, reverse transcription, assay design and validation, and data evaluation, to name a few. Their impact is substantial; more than 18,000 citations of the first MIQE publication (as of December 2025) demonstrate their importance, particularly in the PCR community^[293]^. With the rising popularity of dPCR, the MIQE framework was expanded in 2013^[295]^, revised in 2020^[281]^, and updated in 2025 to reflect technological advances in PCR chemistry and instrumentation^[281,295,296]^. Despite their widespread recognition, many papers reporting qPCR experiments continue to be published without mentioning the MIQE guidelines^[296]^.

Together, the MISEV and MIQE guidelines provide a complementary framework for standardizing EV isolation, characterization, and downstream molecular analysis. Researchers in the EV field are strongly encouraged to follow these guidelines and report RNA quantity and quality, as well as all methodological variables. Integrating EV- specific MISEV standards with molecular assay-focused MIQE standards is essential for strengthening the reproducibility, interpretability, and validity of EV biomarker research^[297]^.

### Biofluid-specific considerations

The ISEV convened a Scientific Reproducibility Task Force to provide tailored recommendations for EV-containing biofluids and biological sources^[298]^. Although universally agreed-upon recommendations have not yet been developed for all possible biological sources of EVs, several task forces have published detailed guidelines for blood, urine, and CSF.

As the most extensively studied biofluid in EV research, blood requires strict control of pre-analytical variables, which are a major source of bias in EV-RNA studies, as recently demonstrated by Dhondt *et al.*^[76,299]^. MISEV2023 provides recommendations for reporting donor characteristics, blood collection methods and collection tubes, post-collection processing, storage conditions, and identification of potential contaminants. The ISEV Blood Task Force additionally released the Minimal Information for Blood EV (MIBlood-EV) reporting tool to harmonize studies involving blood-derived EVs^[78]^.

Because it can be collected non-invasively, urine is the second most studied biofluid in EV research. The ISEV Urine Task Force has developed a position paper outlining the best practices and methodological details for the isolation, normalization, and pre-analytical evaluation of urinary EVs to improve reproducibility and clinical translation^[165]^. Key recommendations include the use of cell-free urine, documentation of both EV isolation and contaminant-depletion methods, and collection of data on both EV and non-EV urinalysis parameters to estimate absolute or relative excretion rates.

CSF is considered a rich source of neurological biomarkers. However, due to the invasive nature of procurement and the limited sample volume and low EV concentrations in valuable CSF samples, special considerations are necessary. Therefore, the ISEV CSF Task Force has provided recommendations regarding best practices for the collection, processing, storage, and reporting of CSF EV studies^[163]^. These recommendations include reporting the anatomic site of collection and volume of CSF drawn, levels of co-isolates/contaminants, the separation and characterization methods used, and whether samples were pooled due to the typically small volume.

### Implementation across the EV-miRNA workflow

Standardization must be consistently applied across all steps of the EV-miRNA workflow, including EV isolation, characterization, RNA extraction, and downstream analysis. The choice of techniques used for EV isolation, separation, and purification depends on the known properties of the specific EV source, the available sample volume, and the desired balance among EV yield, purity, specificity, and integrity. While optimal approaches for each EV source are continually refined in the literature, MISEV2023 provides guidance on commonly used methods for EV preparation, along with typical yield and specificity ranges^[78]^.

As noted above, the guidelines also provide recommendations for EV characterization, including the quantification of particle concentration, size distribution, morphology, and biological composition, such as, proteins, lipids, and RNA. For RNA-focused studies, MISEV recommends reporting assay limitations, including the ability to discriminate between RNA and DNA, limits of detection, and the use of enzymatic pretreatments, such as DNase digestion^[78]^.

With the multitude of isolation techniques available, different combinations of EV and RNA purification methods can yield substantially different sample characteristics and experimental outcomes. Importantly, RNA isolation, storage, and handling should be performed according to both MISEV and MIQE guidelines^[78,261,262,281,283,289,293,295,296]^. Dhondt *et al*. demonstrated that different pre-analytical parameters can substantially alter EV and RNA content^[299]^. For example, consistent processing time following blood collection are critical, although this is not always feasible in clinical settings. For biomarker validation studies, MIQE provides guidelines and checklists specifying the necessary information that should accompany qPCR and dPCR results in publications^[281,293,295,296]^.

## NORMALIZATION STRATEGIES

### EV-level normalization

Several methods can be used to normalize EV preparations based on particle number, protein content, lipid content, or nucleic acid content (see section “RNA ISOLATION FROM EVS”). Such normalization improves comparability across samples from different sources or with varying EV yields^[300]^. Normalization based on particle number can be performed using methods such as NTA and bead-based FCM. The combination of orthogonal methods, including light scattering intensity, fluorescence intensity, and size-based measurements can improve measurement reliability by compensating for complementary principles and technical limitations^[78]^. Depending on the instrument used, NTA can detect both light scattering and fluorescence at various wavelengths^[228]^, although assay-specific detection limits remain incompletely defined and should be empirically determined^[78]^. Normalization of protein content can be achieved using immunoblot analysis of EV-associated markers such as CD9 and CD63^[78,301,302]^ or protein quantification by bicinchoninic acid (BCA)^[302]^. Similarly, the sulfo-phospho-vanillin (SPV) lipid assay quantifies unsaturated lipids for normalization purposes but should be used in combination with particle or protein measurements^[303]^.

Nucleic acids, such as stably expressed reference transcripts, can also be used for EV normalization. For this purpose, dPCR is particularly useful because it enables absolute nucleic acid quantification without requiring standard curves^[281,295,304]^.

### Normalization of RNA, including miRNA

Normalization is also crucial for obtaining reliable results from RNA and miRNA expression analysis. For miRNA expression analysis, precise measurement and normalization using validated stable reference miRNAs are critical, as minor changes can have major cellular consequences^[305,306]^. Stably expressed transcripts can be identified using the geNorm and normFinder algorithms, which calculate stability values for candidate reference genes. Stability values are ranked, with more stable transcripts assigned lower stability values^[307,308]^. Both algorithms are applicable to miRNA reference gene selection^[309,310]^. Another useful tool is the Excel-based BestKeeper software, which allows analysis of up to 10 target genes in addition to housekeeping genes^[311]^. For EV-associated miRNAs obtained from human serum or blood plasma, the online tool and database miREV enables the selection of stable reference genes using six normalization and three stability algorithms^[312]^. Additionally, miREV accounts for tissue-specific expression stability^[312]^. Synthetic spike-ins may be added in equal amounts during RNA isolation or reverse transcription to monitor extraction efficiency, reverse transcription efficiency, and PCR performance across samples^[262]^.

### Reference materials

Light-scattering-based EV detection requires calibration using artificial polystyrene beads, silica beads, or hollow organosilica beads. Although polystyrene and silica beads can be similar in size to EVs, their refractive indices are higher, resulting in greater light-scattering intensities than EVs. Hollow organosilica beads, on the other hand, have a similar refractive index but are monodisperse in size, in contrast to the heterogeneity of EVs^[313]^. In addition to artificial beads, biological reference materials for EVs are available, including viral particles, liposomes, nanoerythrosomes, recombinant EVs, and fluorescently labeled EVs^[313-316]^. The advantages of biological reference materials include their similarity to native EVs in terms of biological composition, size, and refractive index. Additionally, they offer the possibility of adjusting their properties, such as lipid composition, protein content, and RNA content^[313]^.

For miRNA studies, a quality control panel consisting of two sets of synthetic spike-in molecules, including three extraction spike-ins and two RTspike-ins, as well as three endogenous miRNAs, has been proposed for biofluid-derived samples to evaluate RNA isolation yield, RT yield, reverse transcription efficiency, PCR efficiency, and hemolysis^[317]^. However, the RNA-seq workflow still requires optimization before analytical miRNA reference materials that mimic the complexity of biofluids can be developed for diagnostic purposes^[313]^.

## DEVELOPMENT OF miRNA BIOMARKER SIGNATURES

### miRNA biomarker signatures

The relative stability of EV-associated miRNAs, mediated in part by vesicular encapsulation and/or association with ribonucleoprotein complexes makes them attractive candidates for non-invasive biomarker discovery. Several studies have explored the diagnostic potential of EV-associated miRNAs across multiple diseases, particularly cancer. For example, upregulation of miR-421 has been observed in tissue biopsies from patients with gastric cancer^[318]^. In patients with prostate cancer, miR-141 has been consistently upregulated in plasma samples across independent studies^[319,320]^. Furthermore, a specific variant of miR-574-3p, characterized by a 3’ adenosine deletion, was found to be upregulated in the serum of patients with esophageal squamous cell carcinoma^[321]^. Collectively, these findings demonstrate the clinical relevance of EV-associated miRNAs as accessible and stable potential biomarkers for cancer detection and classification.

While individual miRNAs can provide diagnostic information, panels of multiple biomarkers, referred to as biomarker signatures, generally offer greater specificity and reliability. This is particularly important given the limited repertoire of human miRNAs and the fact that each miRNA can regulate numerous target genes^[322]^, indicating that individual miRNAs may have limited disease specificity when used alone. In contrast, integrating multiple miRNAs into a composite biomarker signature enables the identification of disease-associated expression patterns and may reflect coordinated dysregulation of biological pathways relevant to disease pathogenesis. This approach has led to the development of the GASTROClear test for the early detection of gastric cancer, which has received regulatory approval in Singapore, CE certification for *in vitro* diagnostic (IVD) use in the European Union, and FDA Breakthrough Device Designation^[323,324]^. GASTROClear measures 12 circulating miRNAs in blood and achieved a sensitivity of 87% and specificity of 68.4% in a large-scale validation study involving more than 5,000 participants^[325]^. Another important example is the Italian miR-Test for lung cancer detection, which incorporates a panel of 34 miRNAs^[326]^ and has demonstrated robust diagnostic performance, with approximately 75% diagnostic accuracy, sensitivity, and specificity in a validation study involving high-risk individuals^[327]^. Together, these examples demonstrate that liquid biopsy-based miRNA biomarker signatures are progressing from discovery toward clinical implementation.

### Multivariate data analysis

High-dimensional datasets generated from small RNA-seq require analytical approaches that can extract biologically meaningful information from complex gene expression patterns. Therefore, multivariate statistical methods have become indispensable in miRNA biomarker research for identifying relationships among samples, discriminating between biological conditions, and detecting coordinated expression patterns. Techniques such as principal component analysis (PCA), hierarchical clustering (HC), and partial least squares discriminant analysis (PLS-DA) have been widely adopted in transcriptomic studies, including those focusing on EV-miRNAs^[94]^.

Unsupervised approaches are typically used for the initial exploration of datasets. PCA reduces the dimensionality of the miRNA expression matrix while preserving most of its variance^[328]^. When applied to EV-derived miRNA profiles, PCA enables visualization of the similarities and differences among biological groups and helps identify sample outliers, batch effects, and underlying confounding factors. PCA provides an unbiased overview of the dominant sources of variance in the dataset, which is crucial for subsequent refinement of biomarker candidate lists^[329-331]^. Similarly, HC is routinely used to reveal natural clusters in samples or sets of miRNAs. Different algorithms, such as complete linkage, average linkage, and Ward’s method, allow the construction of dendrograms that illustrate the relationships among samples based on their expression patterns^[332]^. HC-derived heatmaps visually represent the differences in expression between groups and help identify clusters of co-regulated miRNAs. These miRNA clusters may correspond to biological pathways or regulatory modules that are consistently perturbed in disease states, thereby providing another approach for biomarker candidate selection^[333]^.

Supervised multivariate approaches, on the other hand, are specifically designed to maximize the separation between predefined classes^[334]^. One example is PLS-DA, which identifies latent components that best capture the variance associated with the disease phenotype. This makes it particularly useful for constructing diagnostic classifiers and evaluating biomarker signatures derived from EV-miRNA sequencing. Importantly, PLS-DA also provides quantitative metrics, such as variable importance in projection (VIP) scores, which rank miRNAs by their contribution to the discriminatory model and can therefore assist in selecting features during biomarker development^[335]^. However, supervised methods must be interpreted with caution to avoid overfitting, especially when the sample size is limited relative to the number of variables, which is a common scenario in miRNA biomarker studies.

Rigorous cross-validation procedures are essential to ensure that the model captures the true biological differences, rather than noise or technical artifacts. This can be achieved, for example, through permutation testing or nested resampling frameworks^[293,329]^. Furthermore, the reproducibility of PLS-DA models should be evaluated by testing their performance on independent datasets or external validation cohorts. Ideally, metrics such as accuracy, sensitivity, specificity, and area under the receiver operating characteristic curve (ROC AUC) are used for this purpose^[336]^.

Overall, multivariate analysis plays a central role in the development of robust EV-miRNA biomarker signatures. Unsupervised techniques enable an unbiased assessment of the data structure and highlight global expression trends. In contrast, ssupervised approaches help construct predictive models that capture disease-specific expression patterns. Moreover, integrated analysis platforms such as caRNAge^[265]^ facilitate the generation of standardized miRNA and miRNA isoform (isomiR) feature matrices, which provide a coherent input structure for downstream multivariate modeling approaches, including PCA, HC, and PLSDA. In combination with rigorous validation strategies, these analytical tools contribute significantly to identifying stable, biologically relevant, and diagnostically useful miRNA signatures.

### miRNA isoforms

Beyond the canonical mature miRNA sequences annotated in reference databases, high-throughput small RNA-seq has revealed a vast diversity of miRNA isoforms, or isomiRs, that arise from imprecise Drosha and/or Dicer cleavage, post-transcriptional nucleotide modifications, or templated and non-templated additions at the 3’ end. These isoforms are not merely rare exceptions but instead represent a pervasive and biologically regulated layer of small RNA complexity. In many tissues and biofluids, the abundance of isomiRs from a given locus can exceed that of the corresponding canonical miRNA. Moreover, in some cases, the dominant biologically active species is an isomiR, rather than the reference sequence. This diversity has important implications for miRNA quantification, EV-mediated communication, and biomarker discovery^[337,338]^.

IsomiRs are broadly categorized into 5’ isomiRs, 3’ isomiRs, and non-templated addition isomiRs, each generated by distinct mechanistic pathways. 5’ isomiRs often result from alternative Drosha or Dicer cleavage positions, producing shifts in the seed region - the 2-8 nt sequence that largely determines miRNA target specificity. Even a single-nucleotide 5’ trimming or extension of the miRNA sequence can alter the predicted targetome, generating an isoform with a unique biological function that is sometimes distinct from, or even antagonistic to, the canonical miRNA. 3’ isomiRs are more abundant than their 5’ counterparts and typically arise from exonuclease trimming or Dicer imprecision. Although the seed region is not altered in 3’ isomiRs, they can modulate AGO loading, miRNA stability, target affinity, and mRNA decay kinetics*.* Non-templated additions, most commonly involving adenylation or uridylation, are thought to influence miRNA stabilization, turnover, and/or selective incorporation into EVs. For example, 3’ uridylation has been associated with enhanced sorting into exosomes and can alter recognition by RNA-binding proteins^[52,339]^.

From a methodological perspective, accurately quantifying isomiRs remains a significant challenge. Conventional RT-qPCR assays and microarrays generally lack the sequence discrimination necessary to resolve isoforms that differ by one or two nucleotides. Many RNA-seq pipelines collapse isomiRs into canonical miRNAs during mapping, thereby discarding biologically relevant information. Therefore, specialized small RNA aligners and isoform-aware quantification tools are required for rigorous isomiR characterization^[265]^. Failure to distinguish between isoforms can lead to misinterpretation of differential expression results, obscure meaningful biomarker signals, and distort apparent variability between biological conditions. This is particularly relevant in EV studies, where sequencing depth and RNA input are typically limited, making precise isoform-level analysis essential. In the context of EV biology, isomiR signatures may provide enhanced granularity for cell-of-origin inference and disease classification. Incorporating isomiR-level data into biomarker development workflows has the potential to substantially improve diagnostic accuracy. Isoform-specific differential expression often shows larger effect sizes and better group discrimination than canonical miRNA-level analyses. The integration of isomiR features into multivariate classifiers, such as PCA, PLS-DA, or sparse PLS-DA (sPLS-DA), can therefore increase model robustness and resolve biological heterogeneity that would otherwise remain undetected. Moreover, disease-associated biases in Drosha or Dicer cleavage and terminal modification patterns may serve as mechanistic biomarkers that reflect altered RNA processing pathways^[340]^. Taken together, the rapidly expanding field of isomiR biology underscores the need for isoform-resolved quantification in comprehensive analyses of miRNA dynamics, particularly in EV-based translational studies.

### Validation of miRNA biomarker expression

Biomarker signatures identified through NGS should be validated using orthogonal analytical methods and, ideally, evaluated in independent cohorts with larger sample sizes. This helps ensure that the signature is not driven by potential biases, cohort-specific effects, or technical artifacts. RT-qPCR is commonly used for such validation due to its high sensitivity, robustness, and cost-effectiveness. However, qPCR primers are typically approximately 20 nt in length, which is nearly the same length as a mature miRNA (~22 nt). Therefore, measuring miRNAs using conventional qPCR primers is not feasible, and, as such, specialized methods have been developed to overcome this limitation. Common techniques include elongation of the mature miRNA during cDNA synthesis with stem-loop primers^[341]^ or two-tailed hairpin primers^[342]^, polyadenylation of the miRNA^[343]^, or the use of locked nucleic acid-based RT-qPCR^[344]^. If the biomarker candidate is a precursor miRNA, assays can be designed to target precursor-specific sequences, including the hairpin structure^[345]^.

For any gene expression RT-qPCR experiment, it is essential to choose one or more reference genes that are stably expressed across all conditions evaluated in the study, including control, disease, and/or treatment groups. As discussed in Section “NORMALIZATION STRATEGIES”, such stably expressed miRNA candidates can be identified from NGS data; for example, algorithms such as geNorm or Normfinder can help select the most stable reference miRNA^[307,308]^. Additionally, all PCR assays must be well-characterized and transparently reported, including data on assay efficiency. Adherence to the MIQE guidelines is strongly recommended (see section “STANDARDIZATION IN EV-miRNA BIOMARKER STUDIES”)^[281,293,295,296]^.

Validation of the candidate miRNA biomarkers is then typically performed by comparing the expression patterns obtained using optimized and validated RT-qPCR assays with those observed in the initial NGS analysis. Finally, the discriminatory power of the final biomarker signature can be assessed using statistical methods such as PLS-DA combined with ROC AUC analysis.

### *In silico* validation of miRNAs

*In silico* validation is a critical step in miRNA research, serving as a computational bridge between sequencing-based discovery and experimental confirmation. Because individual miRNAs exert their biological effects through the coordinated regulation of gene networks rather than single targets, computational analyses must integrate diverse data layers. These include miRNA-mRNA interaction predictions, experimentally supported binding evidence, target gene co-expression patterns, and pathway-level enrichment metrics. This systems-level perspective is particularly important in studies involving EV-derived small RNAs, in which context-specific regulatory circuits may differ substantially from known intracellular miRNA functions.

Target prediction typically begins with queries in established databases, such as TargetScan^[346]^, miRDB^[347]^, and miRTarBase^[348]^. These platforms employ complementary inference strategies, ranging from seed-sequence conservation and thermodynamic stability to machine-learning classifiers trained on Argonaute crosslinking immunoprecipitation sequencing (AGO-CLIP) datasets. Consensus intersection approaches are particularly useful because they increase specificity by retaining interactions supported by multiple independent prediction models rather than relying on a single algorithm, although this approach may reduce sensitivity by excluding context-specific targets supported by fewer resources. For EV-associated miRNAs, incorporating tissue- or biofluid-specific expression constraints can further refine target lists by eliminating contextually irrelevant transcripts^[349]^.

Once high-confidence target sets are established, functional interpretation typically proceeds through pathway enrichment analyses. Platforms such as caRNAge^[265]^, which incorporate bias detection and isoform-level miRNA annotation, provide a stronger foundation for this purpose by ensuring that enrichment is performed on biologically accurate, context-relevant feature sets. Gene Ontology (GO) annotation^[350]^, KEGG analysis^[351]^, and Reactome pathway mapping^[352]^, as well as network-centric frameworks such as STRING^[353]^, assist in the systematic discovery of biological themes underlying miRNA activity. Over-representation analysis (ORA) and gene set enrichment analysis (GSEA) approaches offer complementary advantages. ORA highlights pathways that are disproportionately represented among predicted targets, whereas GSEA evaluates coordinated shifts in ranked gene lists, making it well suited to detecting the subtle regulatory effects typical of miRNA-mRNA interactions^[354,355]^. Enrichment of pathways related to immune regulation, apoptosis, vesicular trafficking, or metabolic rewiring can provide mechanistic insight into how miRNA dysregulation contributes to pathological phenotypes in disease-focused investigations^[356]^.

Critically, integrating pathway analysis with clinical metadata strengthens translational relevance. Correlating miRNA expression or isomiR signatures with patient traits, such as disease stage, survival, and therapeutic response, enables the identification of regulatory pathways most closely associated with clinical outcomes. Multivariate modeling frameworks, including PCA, PLS-DA, network-regularized regression, and Bayesian pathway scoring, can combine miRNA features with pathway-level summaries to enhance predictive performance while reducing dimensionality. This layered approach not only improves biomarker robustness but also highlights the biological processes that are most consistently perturbed across patient cohorts. In the context of EV-derived miRNAs, where cargo selection, release mechanisms, and recipient cell responses form a highly regulated, context-dependent system, *in silico* validation is essential for deciphering the complex regulatory architectures that underlie their diagnostic and therapeutic potential.

## CONCLUSION AND OUTLOOK

Today, miRNAs are regarded as promising circulating EV-associated biomarkers because of their remarkable stability and capacity to regulate disease-associated gene expression programs. The encapsulation of RNA within the EV lipid bilayer and/or association with the EV corona, protects RNA molecules from RNase activity and other degradative processes, enabling their reliable detection in a wide range of biofluids through minimally invasive liquid biopsy approaches. Following sample collection, EVs and their RNA cargo can be isolated using diverse methodologies, and numerous studies have identified disease-specific EV-miRNA signatures. These findings collectively reinforce the concept that EV-miRNAs are robust biomarkers that combine molecular specificity with enhanced stability and partial cell-type and tissue specificity conferred by vesicular packaging. Accumulating evidence across cancer, inflammatory, and cardiovascular diseases indicates that EV-miRNA signatures can complement, and in some cases outperform, conventional blood biomarkers for diagnosis, risk stratification, and treatment monitoring in minimally invasive liquid biopsies^[357,358]^. Despite these advances, translating EV-miRNA biomarkers into routine clinical practice remains a significant challenge. A major limitation of the field is methodological heterogeneity, including the lack of standardized protocols for EV isolation, characterization, and miRNA quantification. Pre-analytical variability, differences in sample type and handling, storage conditions, extraction efficiency, and normalization strategy all contribute to inter-study variability and complicate cross-cohort comparisons^[297,359]^. These technical and biological confounders currently hinder reproducibility and delay the regulatory approval and clinical implementation of EV-miRNA-based diagnostic assays^[360-362]^. In parallel, emerging conceptual frameworks, such as the nasopharyngeal carcinoma ecology theory^[363]^, emphasize the need to move beyond static biomarker assessment toward dynamic, systems-level monitoring of the disease progression. Tumors are increasingly understood as evolving biological systems shaped by heterogeneous cell populations and their microenvironments. In this context, serial liquid biopsy sampling combined with multiparametric analysis of EV-miRNAs, circulating tumor cells (CTCs), and complementary imaging offers the potential to capture temporal disease dynamics. Such approaches may enable earlier relapse detection, improved monitoring of minimal residual disease, and implementation of adaptive biomarker-guided treatment strategies. Consequently, shifts in EV-miRNA profiles or CTC phenotypes can inform timely therapeutic interventions, including treatment escalation, treatment de-escalation, or microenvironment-targeted approaches, such as niche disruption.

To address these limitations, adherence to international guidelines and position papers is essential. Frameworks such as MIQE and MISEV play critical roles in improving transparency, reproducibility, and methodological rigor in EV and miRNA research. Integrating these frameworks into research workflows and clinical diagnostic practices may improve the quantitative accuracy of biomarker analyses and, consequently, their overall validity and scientific credibility^[297,360]^. Although implementation requires substantial resources, including standardized workflows, specialized instrumentation, and comprehensive reporting practices, these efforts are necessary to ensure data reliability and the biological validity. Standardization is crucial for enabling longitudinal analyses and establishing clinically actionable thresholds.

In conclusion, circulating EV-miRNAs offer key advantages through their ability to provide diagnostic and prognostic information that complements imaging and conventional laboratory testing. Their integration into multiparametric biomarker panels can improve sensitivity and specificity, particularly for complex diseases. In oncology and cardiology, specific EV-miRNA signatures have been linked to early disease detection, prognosis, and treatment response, underscoring their value in longitudinal patient monitoring and clinical decision-making. While EV-associated miRNAs represent a powerful and versatile class of biomarkers with clear potential to transform non-invasive diagnostics and personalized medicine, their successful clinical translation will depend on the convergence of robust standardization, careful biofluid selection, and the adoption of dynamic, longitudinal monitoring strategies. By aligning technological advances with harmonized methodological frameworks, the field is well positioned to move from exploratory research toward clinically actionable EV-miRNA-based diagnostics that can meaningfully improve patient outcomes.

## DECLARATIONS

### Copyright

© The Author(s) 2026.
